# Triage human papillomavirus testing for cytology-based cervical screening in women of different ages in primary hospitals: A retrospective clinical study

**DOI:** 10.1097/MD.0000000000022320

**Published:** 2020-09-18

**Authors:** Yuan-yuan Zhang, Xiao-qin Xu, Dan Zhang, Jie Wu, Hong-xiu Zhang

**Affiliations:** a Department of Obstetrics and Gynecology, the First Affiliated Hospital of Nanjing Medical University; b Department of Obstetrics and Gynecology, Gulou District Phoenix Street Community Health Service Center, Nanjing; c Department of Obstetrics and Gynecology, Dianshan Lake People's Hospital of Kunshan City in Jiangsu province, Kunshan, Jiangsu, China.

**Keywords:** cervical cancer screening, human papillomavirus test, thin-prep cytologic test

## Abstract

Cervical cancer is a serious global health problem. The objective of this study was to provide a suitable cytology-based cervical screening method in women of different ages in primary hospitals.

This study was a retrospective cohort study that included 9765 women who underwent primary cytology-based cervical screening and were grouped by age (35–44, 45–54, and 55–64 years old). Patients with abnormal cytology on the primary cervical thin-prep cytologic test (TCT) were advised to undergo triage human papillomavirus (HPV) test. Furthermore, patients with positive outcomes of the 2 indices underwent cervical tissue biopsy. The positive rate of TCT and HPV was compared among the 3 defined age groups. The sensitivity, specificity, and positive predictive value of TCT and HPV were assessed.

In total, 2.5% (241/9765) of women had atypical squamous cells of undetermined significance or worse by TCT. High-risk (HR)-HPV infection was found in 70 triage participants. Neoplastic changes were confirmed in 95 patients (95/437, 21.7%) by biopsies. Among the different age groups, the positive rate of abnormal cytology was significantly different (*P* = .003), and the positive rate of HR-HPV was similar (*P* = .299). The sensitivity of initial TCT testing to detect intraepithelial neoplasia was higher than that of triage HPV testing, whereas the specificity, the positive predictive value of triage HPV testing was higher than that of TCT. The Youden index of HPV testing was higher than that of TCT detection in the 3 age groups, namely 0.582 versus 0.432, 0.553 versus 0.228, and 0.416 versus 0.332, respectively.

The results of this study indicate that TCT testing is suitable as a cervical cancer screening method for women ≥35 years old in primary hospitals. Triage testing for women with HR-HPV has a high negative predictive value, reduces the rate of misdiagnosis, seems to be an excellent triage method for repeat atypical squamous cells of undetermined significance, and reduces the number of referral colposcopies preventing unnecessary overtreatment. The results of this study provide a crucial foundation for a unified guideline cervical cancer screening for primary health care institutions.

## 1 Introduction

Cervical cancer is the fourth most common cancer of women worldwide.^[1]^ According to the National Central Cancer Registry of China in 2015, the incidence of cervical cancer was 98.9 per 100,000 women, and the mortality rate was 30.5 per 100,000 women.^[2]^ In contrast to the declining trend of cervical cancer incidence in developed countries, the incidence of cervical cancer in China has significantly increased. Hence cervical cancer is still one of the main threats to women's health in China.

Cervical screening has been implemented in some rural and urban areas in women aged 35 to 59 and 35 to 64 years since 2009.^[3]^ The on-going policy for cervical screening is to perform cytology-based cervical tests for women every 3 years within primary care. However, in primary hospitals, the detection of high-risk human papillomavirus (HR-HPV) is not widely implemented. It is recommended that patients with abnormal cytology undergo a cervical biopsy. Cervical cancer screening work throughout China has not been evenly distributed, with various regions having different conditions. Nevertheless, HR-HPV screening has been implemented as the triage of equivocal cytological results in some places.

With the improvement of screening technologies, cervical pap smear, and visual inspection with acetic acid and Lugol's iodine are currently only used in remote and economically deficient areas in China. Thin-prep cytologic Test (TCT) screening has become the primary screening method in China.^[4,5]^ However, the TCT has low predictive value in detecting high-grade cervical cancer precursors, as its accuracy depends on sample acquisition, as well as the skill and experience of the physician.^[6]^

Many studies have shown that most invasive cervical cancers (ICCs) are caused by HR-HPV infection.^[7,8]^ The persistence of HR-HPV infection in epithelial cells of the cervix is essential to progression towards high-grade cervical disease and ICC.^[9]^ Without treatment, the risk of ICC increases with an increasing degree of dysplasia by affecting about 50% of women with cervical intraepithelial neoplasia grade 3 (CIN3).^[10]^ Human papillomavirus (HPV) screening techniques have the advantages of good reproducibility, easy training, and high sensitivity. Recent findings have suggested that HPV DNA detection on self-collected cells has advantages.^[11]^ TCT, combined with HPV detection, significantly improves the screening sensitivity and specificity for cervical cancers. However, it may not be suitable for women in primary hospitals because it is expensive. Therefore, the value of TCT and/or HR-HPV testing for primary screening is mostly unknown. There is also a lack of evidence and reliable data on the differences in the efficacy of TCT and/or HR-HPV detection methods in women of different ages.

Therefore, it is of great importance to find a screening program that has specific sensitivity and specificity in primary hospitals. The objective of this study was to compare and evaluate the efficacy of TCT and HR-HPV screening methods for cervical cancer in adults of different ages to find an optimal screening method that can improve the sensitivity and specificity of detection, save medical costs, and reduce overdiagnosis and treatment for women in primary health care institutions.

## 2 Materials and methods

### 2.1 Subjects and sample collection

**Figure 1:**
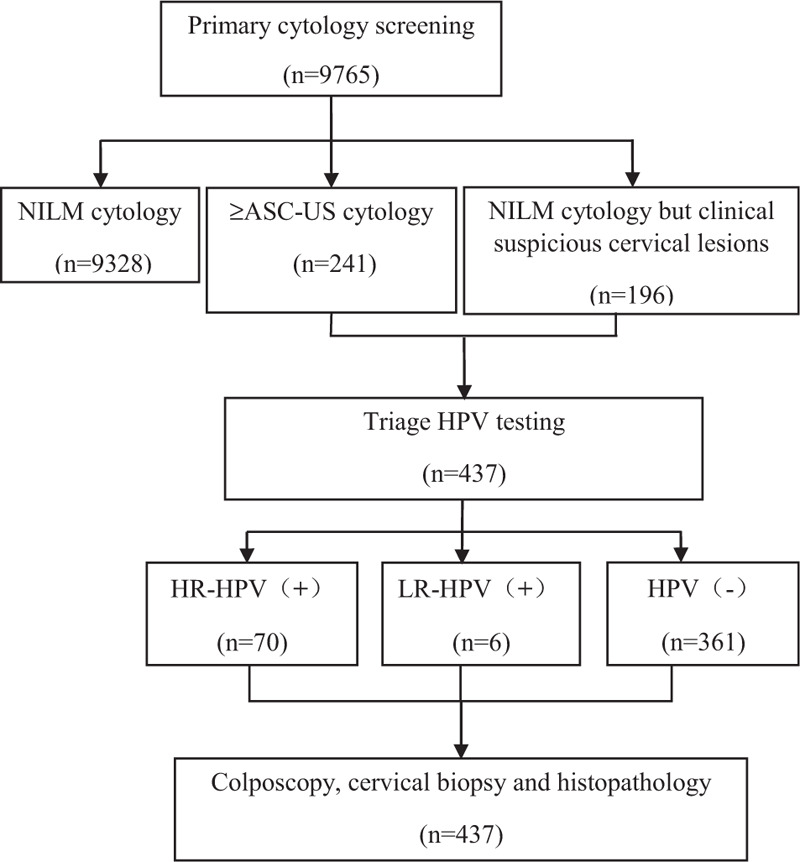
Cervical screening flowchart in this research. ASC-US = atypical squamous cells of undetermined significance, HPV = human papillomavirus, HR-HPV = high-risk HPV, LR-HPV = low-risk HPV, NILM = no intraepithelial lesions or malignancy.

This was a retrospective cohort study that targeted women who underwent cervical cancer and precancerous lesion screening at 3 primary hospitals from December 2017 to December 2018. The 3 primary hospitals included 2 community hospitals (Gulou District Phoenix Street Community Health Service Center and Ninghai Street Community Health Service Center, Nanjing, Jiangsu Province, China) and 1 township hospital (Dianshan Lake People's Hospital of Kunshan City, Suzhou, Jiangsu Province, China). The Medical Ethics committee approved the survey of the First Affiliated Hospital of Nanjing Medical University. Informed consent was obtained from all individual participants. The inclusion criteria for participants were:

1) patients with rural household or urban household registration;
2) patients who were not pregnant or puerperal;
3) patients who were not having a menstrual period; and
4) patients voluntarily participated in the physical examination. Patients with acute genital inflammation, or severe other diseases of the genital tract, or had a history of cervical or total uterus resection were excluded. The cervical cancer screening examination included a gynecological examination, primary cytology screening, triage HPV testing, and colposcopy with cervical biopsy. The screening flowchart is shown in Figure 1.

### 2.2 Cervical TCT

The participants were asked to refrain from vaginal lavage or sexual intercourse for 48 hours before the examination. Cervical TCT was performed after routine gynecological examinations. Patients adopted the bladder lithotomy position and exposed the cervix. Cervical secretions were wiped with a sterile cotton ball. Then, a special cervical brush was gently inserted into the inner cervix (1 cm), and the cervical brush was rotated 3 to 5 times clockwise to collect the cervical cells. Next, the brush was placed in the cell preservation solution. Then the specimens were prepared at the Inspection Agency, and tablets for TCT microscopic examination were read. The cytology results were reported using the Bethesda System terminology.^[12]^ The grade categories of the results from low to high severity in order were:

1) no intraepithelial lesion or malignancy (NILM);
2) atypical squamous cells of undetermined significance (ASC-US);
3) ASC that cannot exclude a high-grade lesion (ASC-H);
4) low-grade squamous intraepithelial lesions (LSIL);
5) high-grade squamous intraepithelial lesions (HSIL); and
6) squamous cell carcinoma or cervical adenocarcinoma. ASC-US and more severe grades were considered abnormal cytology results.

### 2.3 HPV DNA test

After the TCT, women with abnormal cytology results were advised to undergo triage HPV DNA testing as an adjunct to the cytology test. Some patients whose TCT results showed NILM but presented with clinically observed cervical contact bleeding, cervical ectropion, or other suspected cervical lesions were also advised to undergo HPV DNA testing (Fig. 1). The cervical exfoliated cells collected before the operation were genotyped for HPV. A Cytobrush was inserted into the endocervical canal until the brush bent against the ectocervix. Then the cervical brush was rotated clockwise 5 times. After the specimens were collected, the brush head was placed into a collection vial that was labeled with the patient's name, the collection date, and patient number before the HPV types were checked. All specimens were sent to our central laboratory within 1 week of collection for the determination of the HPV genotype. The Inspection Agency conducted HPV DNA extraction, PCR amplification, hybridization, membrane washing, and color development. HPV DNA typing was detected by real-time fluorescent PCR and hybridization capture (YN-H16; Yaneng Biotechnology Co., Ltd., Shenzhen, China) including 17 high-risk or putative high-risk types (16, 18, 31, 33, 35, 39, 45, 5l, 52, 53, 56, 58, 59, 66, 68, 73, and 82) and 6 low-risk types (6, 11, 42, 43, 81, and 83). The detection of high-risk or low-risk subtypes was considered a positive result.

### 2.4 Histopathological examination

Women with a positive outcome of any of the 2 indices underwent colposcopy with biopsies and/or endocervical curettage (ECC). All cervical biopsies were performed under direct vision by colposcopy. Specimens were obtained from the area with the most severe dysplastic cervical lesions according to visual examination and colposcopy with VIA + VILI. Four-quadrant cervical biopsies were taken by colposcopy if the lesion area was not found. The specimens were routinely fixed in formaldehyde. The pathological results of cervical tissue were used as the gold standard for diagnosis. The diagnosis was divided into:

1) normal/inflammation;
2) cervical intraepithelial neoplasia grade 1, cervical intraepithelial neoplasia grade 2 (CIN2), CIN3; and
3) invasive carcinoma. Histopathological examination results were used to identify neoplastic changes and other infections with a positive diagnosis referring to cervical intraepithelial neoplasia grade 1 and more severe lesions (CIN1+) and a negative diagnosis referring to normal/inflammation.

### 2.5 Statistical analysis

All the original data were carefully checked and recorded. Statistical analysis was performed using SPSS 20.0 (IBM, Armonk, NY). The association between the categorical variables was analyzed using the Chi-squared test. The pathological test results were used as the gold standard. The receiver operating characteristic curve and Youden index was used to evaluate the diagnostic value of TCT and HPV on positive screening results by age group. The area under the curve (AUC) = 0.5–0.7 was defined as low accuracy, AUC = 0.7–0.9 was defined as moderate accuracy, and AUC > 0.9 was defined as high accuracy. *P* < .05 was considered statistically significant.

## 3 Results

A total of 9765 women aged 35 to 64 years were included in this study (Table 1). They were divided into 3 groups according to age: 35 to 44 (n = 3211), 45 to 54 (n = 4101), and 55 to 64 (n = 2453) years old.

**Table 1:**
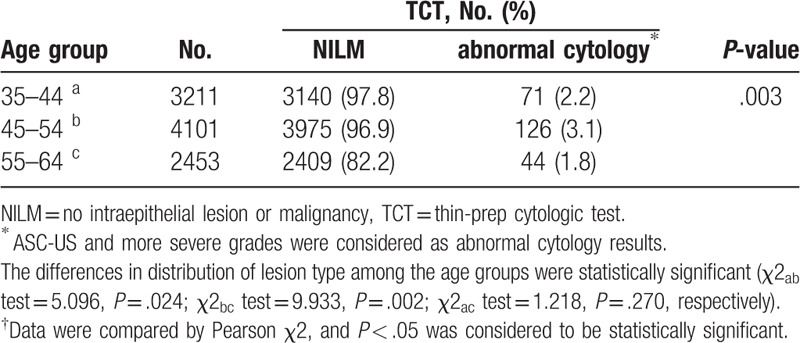
Comparison of thin-prep cytologic test examination results in different age groups.

### 3.1 Cervical TCT results among different age groups

Under the TCT examination, 9524 (97.5%) had NILM, and 241 (2.5%) had abnormal cervical cytology (Fig. 1). Abnormal cytology was found in 71 (2.2%) women in the 35- to 44-year group, 126 (3.1%) in the 45- to 54- year group, and 44 (1.8%) in the 55- to 64-year group (*P* = .003). Between the groups of 35 to 44 and 45 to 54 years, the difference was statistically significant (*P* = .024). Similarly, between the groups of 45 to 54 and 55 to 64 years, the difference was statistically significant (*P* = .002). However, no statistically significant difference was detected between the 35 to 44 and 55 to 64-year age groups (*P* = .270) (Table 1).

### 3.2 HPV-DNA typing test results among different age groups

Patients with abnormal cytology (n = 241) and normal cytology but clinical suspicious cervical lesions (n = 196) were advised to undergo triage HPV DNA testing. Valid results for both cytology and HPV were obtained for 437 women (Table 2). Among the women with normal cytology, the prevalence of HR-HPV and LR-HPV infection were 4.1% and 0.5%, respectively. Among women with abnormal cytology, 25.7% (62/241) had HR-HPV infection, and 2.1% (5/241) had LR-HPV infection. Among the 67 HPV positive women with abnormal cytology, 48 had ASC-US, 6 had ASC-H, 2 had HSIL, and 11 had LSIL. Five of the women with LR-HPV infection had ASC-US. The proportion of having HR-HPV statistically differed between women with abnormal cytology and normal cytology significantly (*P* < .001) (Table 2), but with no significant difference among the different age groups (*P* = .299) (Table 3).

**Table 2:**
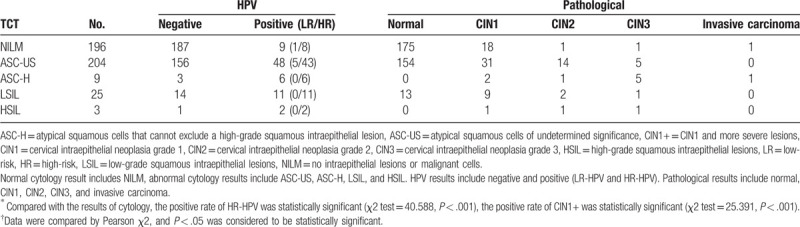
Comparison of human papillomavirus and pathological results with cytology results^∗^.

**Table 3:**
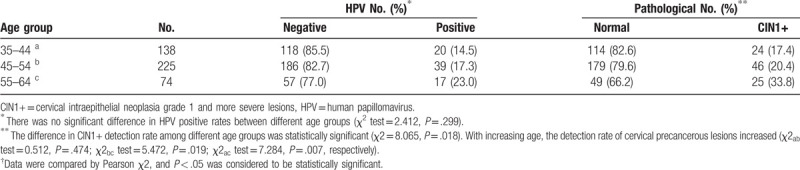
Comparison of human papillomavirusdetection and pathological results in different age groups.

### 3.3 Histopathological results among different age groups

Among the 9765 women, 437 underwent colposcopy with biopsies and/or ECC. Normal or inflammatory results were obtained in 342 (78.26%) women, cervical intraepithelial neoplasia grade 1 in 61 (13.96%), CIN2 in 19 (4.35%), CIN3 in 13 (2.97%), and invasive cervical carcinoma in 2 (0.46%). The detection rate of CIN1^+^ was 21.7% (95/437). The results showed that the 55- to 64-year age group had the highest rate of CIN1+ detection (33.8%, 25/74), followed by the 45- to 54-year age group (20.4%, 46/225), whereas the 35- to 44-year age group had the lowest detection rate (17.4%, 24/138). The difference between the various age groups was statistically significant (*P* = .018). With increasing age, the detection rate of cervical precancerous lesions increased (Table 3).

### 3.4 Accuracy evaluation of TCT and HPV DNA test

AUC across all age groups was 0.70 to 0.90, indicating that the accuracy of the 2 screening methods for diagnosis CIN1^+^ was moderate (Table 4). The highest sensitivity and specificity for TCT and HPV DNA were all in the 35- to 44-year age group. For TCT, the highest sensitivity and specificity was 87.0% (95% CI: 73.2%–100%) and 56.3% (95% CI: 47.1%–65.4%), respectively. While for HPV DNA, the highest sensitivity and specificity was 60.9% (95% CI: 40.9%–80.8%) and 97.3% (95% CI: 94.3%–100%), respectively. The lowest sensitivity and specificity for TCT was 73.9% (95% CI: 61.2%–86.6%) and 48.9% (95% CI: 41.5%–56.2%) in the 45- to 54-year age group, respectively. While the lowest sensitivity and specificity for HPV DNA was 48.0% (95% CI: 28.4%–67.6%) and 93.6% (95% CI: 86.6%–100%) in the 55- to 64-year age group, respectively. The Youden index of HPV testing was higher than that of TCT detection in the different age groups (0.582 vs 0.432, 0.553 vs 0.228, 0.416 vs 0.332, respectively) (Table 5).

**Table 4:**
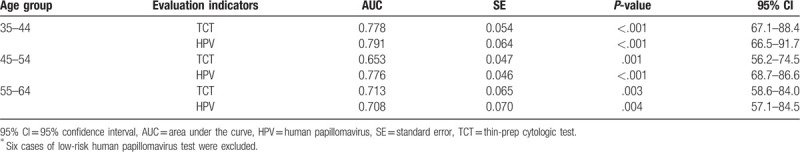
Area under the curve of thin-prep cytologic test and human papillomavirus test tests for the diagnosis of cervical intraepithelial neoplasia grade1 and more severe lesions in different age groups^∗^.

**Table 5:**
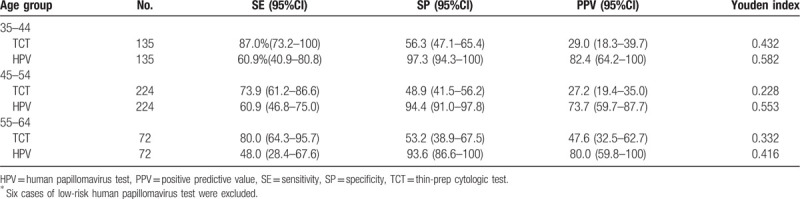
Diagnosis-related evaluation indicators of 2 diagnostic methods in different age groups^∗^.

## 4 Discussion

Primary hospitals have the main task of national free screening for cervical cancer in China. This study shows the value of detecting cervical cancer and precancerous lesions using primary TCT screening and triage HPV DNA testing in different age groups in the primary care setting. In the present study, we retrospectively analyzed women with abnormal cytological in an organized free cervical cancer screening program based on cytology screening strategy to perform triage HPV-DNA testing before referral to colposcopy to evaluate the value of HPV testing, with the goal of optimizing the strategy of current cervical cancer screening and providing some references. According to previous studies, the detection rate of cervical precancerous lesions increases with increasing age.^[13,14]^ The results of this study showed that there was indeed a difference in the detection rate of cervical precancerous lesions in different age groups. Moreover, it also demonstrated that cervical histopathological results increased with age (*P* = .018).

In the current study, there was a significant difference in abnormal cytology in different age groups (*P* = .003). Cytology is the most frequently used primary screening test due to its better sensitivity. The stages of cervical carcinogenesis include the persistence of HPV infection rather than clearance of the virus, which is linked to the development of a high-grade precursor lesion or “precancer”.^[15]^ Some precancer stages, such as chronic cervicitis, wet squamous changes, and cervical lesions, can be reversed and/or treated if they are detected early enough, which can radically reduce cervical cancer incidence.^[16,17]^ So, HPV is recommended as a triage testing or co-testing for women with cytology. However, in China, TCT and HPV co-testing has only been conducted in a small number of areas in the National Cervical Cancer Screening Program, which does not include Jiangsu Province. With limited financial support, more women can participate in screening, and the benefits may be more significant. The optimal strategy for cervical cancer screening is to expand screening rates to maximize the benefits of screening while minimizing potential hazards. In our study, women with ASC-US were recommended to undergo triage HPV-DNA testing as an adjunct to cytology. Of these, among 67 HPV positive women with abnormal cytology, 43 had ASC-US, 6had ASC-H, 2 had HSIL, and 11 had LSIL. Five of the women with LR-HPV infection had ASC-US. The proportion with HR-HPV did statistically significantly differed between women with abnormal cytology and normal cytology (*P* < .001), with increasing age, and the positive rate of HPV increased, but with no significant difference among the different age groups (*P* = .299). The infection rate of HPV different from the reported by You et al,^[18]^ which was high in the age groups of 26 to 30 and 51 to 55 years, accounting for 87.7% (71/81) and 79.7% (51/64), respectively, while it was lower in the >55 years group at 28.6% (14/54) in Shandong provinces of China; this difference was considered to be related to the age of the population and size of the sample. Due to its relatively high specificity (97.3%, 94.4%, and 93.6%, respectively) and positive predictive value (82.4%, 73.7%, and 80.0%, respectively) in detecting CIN1+ lesions in the present study, HR-HPV seems to be useful in the triage of repeat ASC-US or worse among different age groups, which reduces the rate of misdiagnosis. However, our results should be interpreted with caution because a selection bias cannot be ruled out, due to the fact that the use of HR-HPV test was not systematic in this setting.

For primary hospitals, screening methods should be economical and effective.^[3,19]^ In other words, the screening rate of cervical cancer and precancerous lesions should be increased as much as possible, and the rate of missed diagnosis should be reduced with minimum economic investment.^[20]^ Hence, the sensitivity of screening methods should be increased as much as possible. An excellent screening method should not miss any true-positive patients and also exclude all true-negative patients, limiting the economic, and psychological burden on women.^[21]^

In this study, the sensitivity of TCT detection was higher than that of HPV DNA testing across all age groups. By contrast, the specificity, positive predictive value for triage HPV testing was significantly higher than that of TCT in all age groups. These results suggest that triage TCT testing is successfully able to diagnose patients with cervical lesions as the primary method of secondary prevention of cervical cancer and is suitable for women of all ages. HR-HPV has a better ability to screen people with virtually no cervical lesions as an auxiliary screening, which should reduce the number of referral colposcopies, thereby preventing unnecessary overtreatment. Katki et al^[22]^ reported that cumulative risks for cancer of persistent HPV infection, CIN2, and CIN3 were 56%, 2.8%, and 1.1% while follow-up 1 year, respectively; follow-up 2 years were 37%, 4.8%, and 2.0%.

Furthermore, a meta-analysis based on the results of RCTs found a significantly lower rate of cervical cancer among women with negative HPV testing results.^[23]^ Potential adverse effects of colposcopy procedures related to cervical cancer screening included pain, bleeding, discharge, and anxiety.^[24]^ In the present study, triage HPV testing of repeat abnormal cytology increased its specificity and positive predictive value and reduced the unnecessary injuries to patients.

Some previous studies have shown that HPV-DNA detection is more sensitive but less specific compared with cytology for high-grade CIN.^[25,26]^ However, the present study had some differences. On the 1 hand, the population of HPV testing was limited. The HPV as a triage testing was conducted based on people with abnormal cytology results before colposcopy referral to exclude more low-risk people, thus reducing the chances of invasive surgery and saving medical costs. On the other hand, CIN1^+^ was used as the endpoint of diagnosis to identify more high-risk populations. These factors can help in the follow-up care of high-risk groups, including repeat TCT test after 12 months. HPV testing alone or co-testing is the main difference between the European and American guidelines: the American guidelines recommend co-testing with HPV and cytology,^[27]^ while the European guidelines recommend HPV alone.^[28]^ China has a large population and regional economic distribution. The current cervical cancer screening program in China is implemented in a “3-Step” strategy, including TCT, HPV testing, and colposcopy, and is carried out in a diversified manner, adopting different screening strategies for different groups of people. Opportunistic screening, which aims to screen out abnormal cases at a minimum cost and select the optimal screening method according to local conditions, can bring the most significant benefit to the majority of women at limited medical cost. Therefore, primary hospitals should expand screening coverage of cervical cancer with more options for low-cost screening strategies.

The strength of the present screening strategy was that it was based on an opportunistic screening based approach focusing on a community health care maternal child health system,^[3]^ which is a public health service system run by the government of People's Republic of China. The group was organized by the government in primary medical institutions, with clear screening guidelines. The data provided an accurate indication of the cervical lesions and cancer prevalence in the basic population of the region. The limitation of the present study is relatively small of sample size, which may need further large-scale studies to confirm the conclusion.

## 5 Conclusions

In conclusion, the results of this study indicate that TCT testing is suitable as the primary cervical cancer screening method for women of all ages in primary hospitals. High-risk HPV as triage testing has a high specificity, and positive predictive value, reducing the rate of misdiagnosis, seems to be an excellent triage method for repeat ASC-US, the numbers of referral colposcopies can be reduced to avoid unnecessary overtreatment. At present, there is no unified cervical cancer screening guideline. This study provides a crucial foundation for a unified cervical cancer screening guideline for primary health care institutions.

## Acknowledgments

The authors are thankful to Gilmour Tim for his help in preparing the manuscript.

## Author contributions

Z-YY: study design, literature search, data acquisition, manuscript preparation, and manuscript editing. X-XQ: study design, literature search, data acquisition, statistical analysis, manuscript preparation. ZD: literature search, data acquisition, statistical analysis, manuscript preparation. Z-HX: study design, the definition of intellectual content, and manuscript review. WJ: definition of intellectual content, manuscript review. Hongxiu Zhang orcid:0000-001-5995-6460

**Writing – review & editing:** Hongxiu Zhang.

## Abbreviations

Abbreviations: ASC-H = ASC-US that cannot exclude a high-grade lesion, ASC-US = atypical squamous cells of undetermined significance, AUC = area under the curve, CIN1+ = cervical intraepithelial neoplasia grade1 and more severe lesions, CIN2 = cervical intraepithelial neoplasia grade 2, CIN3 = cervical intraepithelial neoplasia grade 3, HPV = human papillomavirus, HR-HPV = high-risk human papillomavirus, HSIL = high-grade squamous intraepithelial lesions, ICC = invasive cervical cancer, LSIL = low-grade squamous intraepithelial lesions, NILM = no intraepithelial lesion or malignancy, TCT = Thin-Prep Cytologic test.
